# Supplementary material for: Modification of Thermo-Chemical Properties of Hot-Pressed ZrB2-HfB2 Composites by Incorporation of Carbides (SiC, B4C, and WC) or Silicides (MoSi2 and CrSi2) Additives
Source: Materials (Basel). 2025 Aug 11;18(16):3761. doi: 10.3390/ma18163761 (PMC12387152; doi:10.3390/ma18163761)
Supplement: Supplementary file 1 [file materials-18-03761-s001.zip › materials-3763467-supplementary.pdf]

*Supplementary*

# Modification of Thermo-Chemical Properties of Hot-Pressed $ZrB_2$-$HfB_2$ Composites by Incorporation of Carbides (SiC, $B_4C$, WC) or Silicides ($MoSi_2$, $CrSi_2$) Additive

**Agnieszka Gubernat [1], Kamil Kornaus [1], Dariusz Zientara [1], Łukasz Zych [1], Paweł Rutkowski [1], Sebastian Komarek [1], Annamaria Naughton-Duszova [2], Yongsheng Liu [3], Leszek Chlubny [1], Zbigniew Pędzich [1*]**

[1] Faculty of Materials Science and Ceramics, AGH University of Krakow, al. Adama Mickiewicza 30, 30-059 Kraków, Poland; gubernat@agh.edu.pl (A.G.); kornaus@agh.edu.pl (K.K.); zientara@agh.edu.pl (D.Z.); lzych@agh.edu.pl (Ł.Z.); pawelr@agh.edu.pl (P.R.); seko@agh.edu.pl (S.K.); leszek@agh.edu.pl (L.C.)

[2] Institute of Materials Research, Slovak Academy of Sciences, 47 Watsonova St., 040 01 Košice, Slovakia; aduszova@saske.sk

[3] Science and Technology on Thermostructural Composite Materials Laboratory, Northwestern Polytechnical University, Xi'an 710072, China; yongshengliu@nwpu.edu.cn

* Correspondence: pedzich@agh.edu.pl; Tel.: +48-12-617-23-97

## Supplementary Materials

Figure S1 shows XRD patterns of all composites.

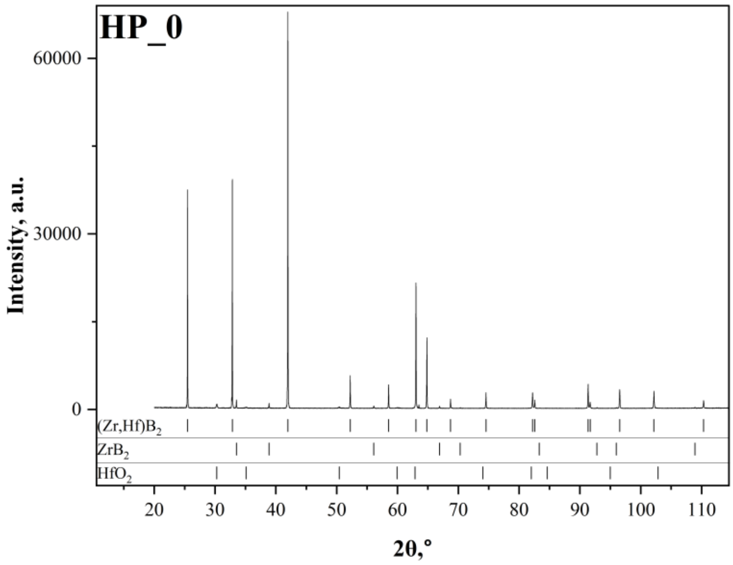

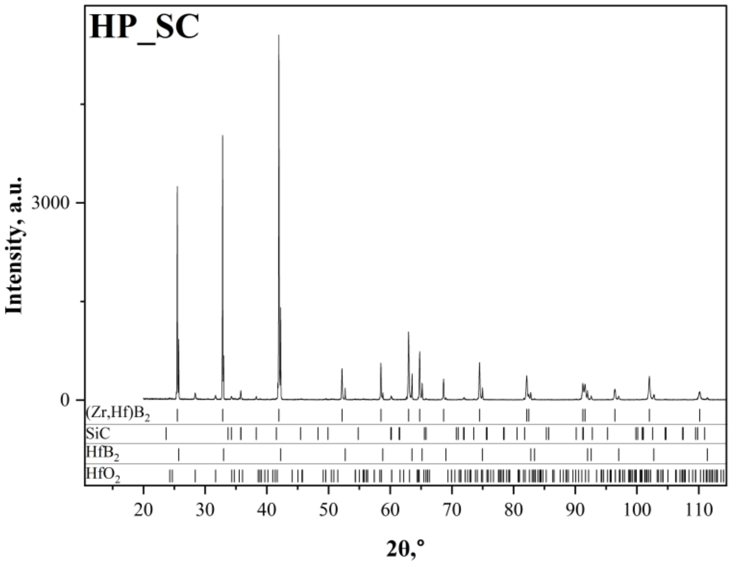
HP_SC
Intensity, a.u.
2θ,°
(Zr,Hf)B$_2$
SiC
HfB$_2$
HfO$_2$

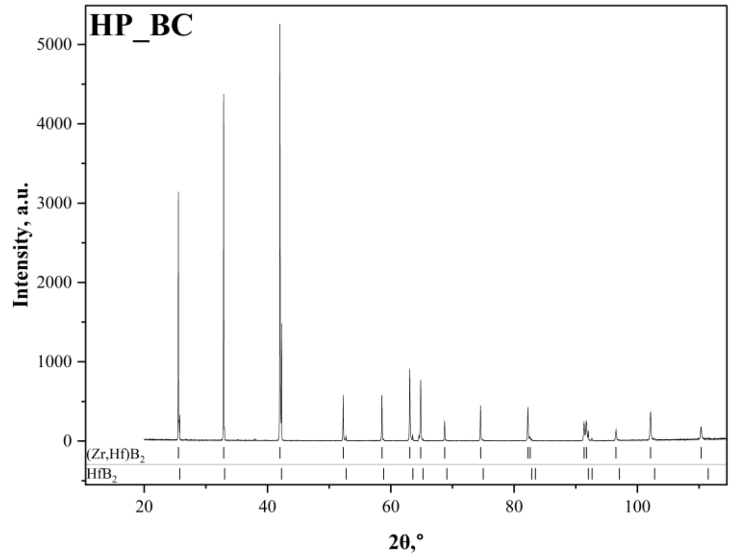
HP_BC
Intensity, a.u.
2θ,°
(Zr,Hf)B$_2$
HfB$_2$

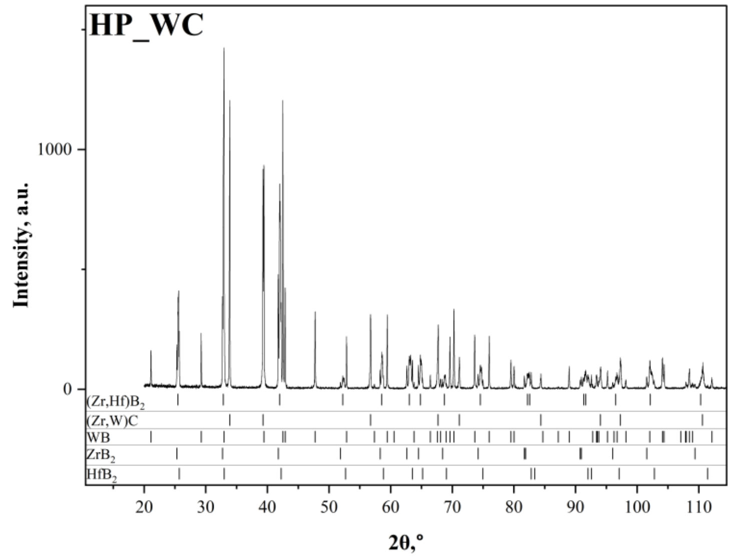
HP_WC
Intensity, a.u.
2θ,°
(Zr,Hf)B$_2$
(Zr,W)C
WB
ZrB$_2$
HfB$_2$

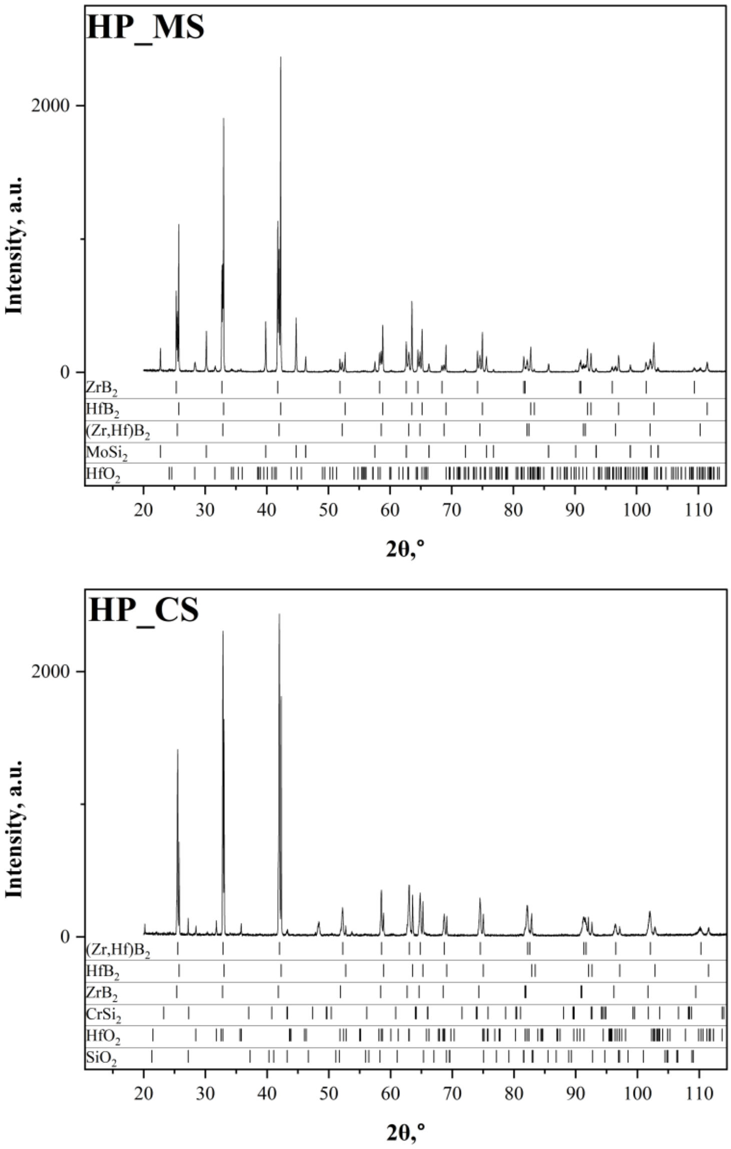


**Figure S1.** XRD patterns of $ZrB_2$-$HfB_2$-MX composites.

Figure S2 shows an SEM image of the HP_BC composite together with an EDS analysis of the chemical composition in the micro-areas. The analysis shows that the darkest areas visible in the micrograph are composed of boron and carbon.

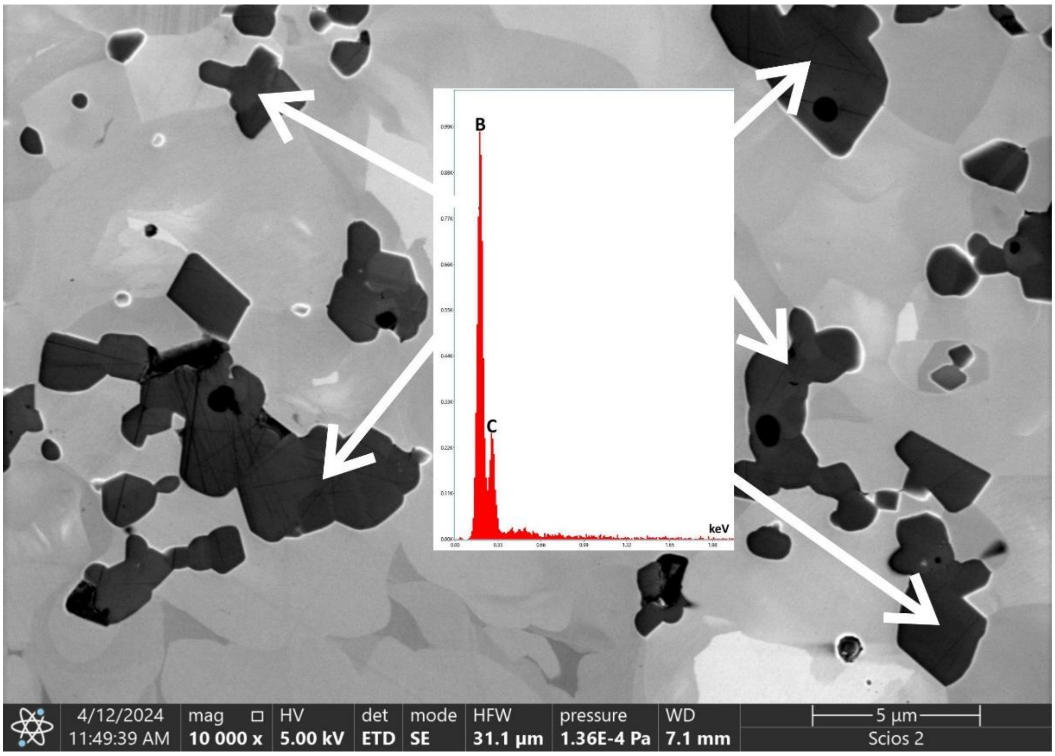


**Figure S2.** SEM microstructure and spot EDS chemical analysis of the HP_BC composite.

Figure S3 presents the results of the XRD phase composition analysis of the HP_BC composite performed using the method with an internal standard. Zinc oxide ZnO in an amount of approximately 15 % was used as the standard. The results of the analysis show that significant amounts of amorphous phase are present in the HP_BC composite. This phase is observed in the SEM images as the darkest boron-rich areas. The roentgenogram shows impurities in the form of $SiO_2$ and $Al_2O_3$, which originate from the mortar in which the sample was crushed.

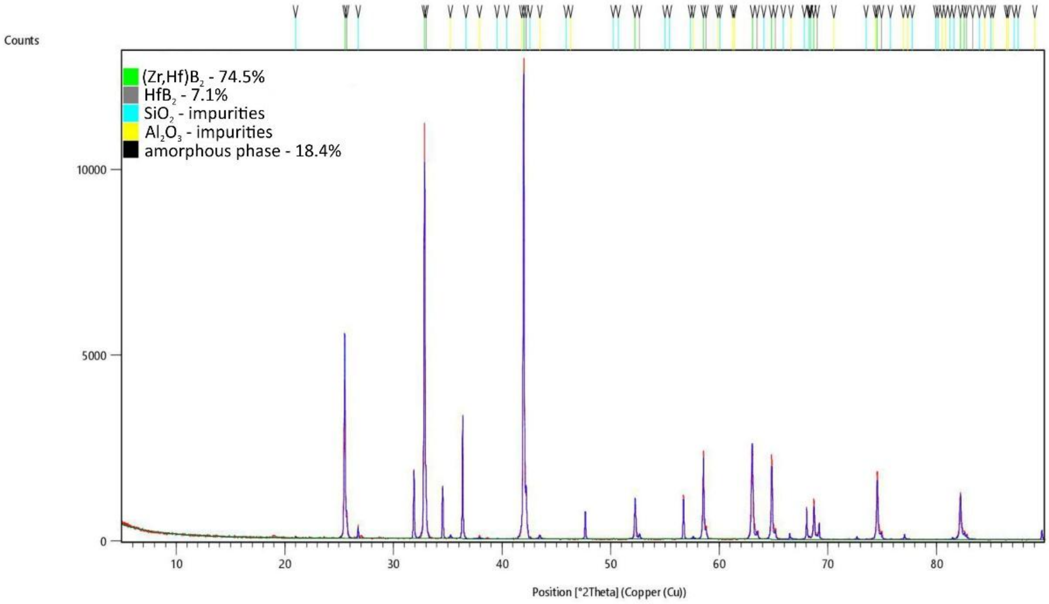


**Figure S3.** Results of qualitative and quantitative analysis of the phase composition of the HP_BC composite with ZnO standard.

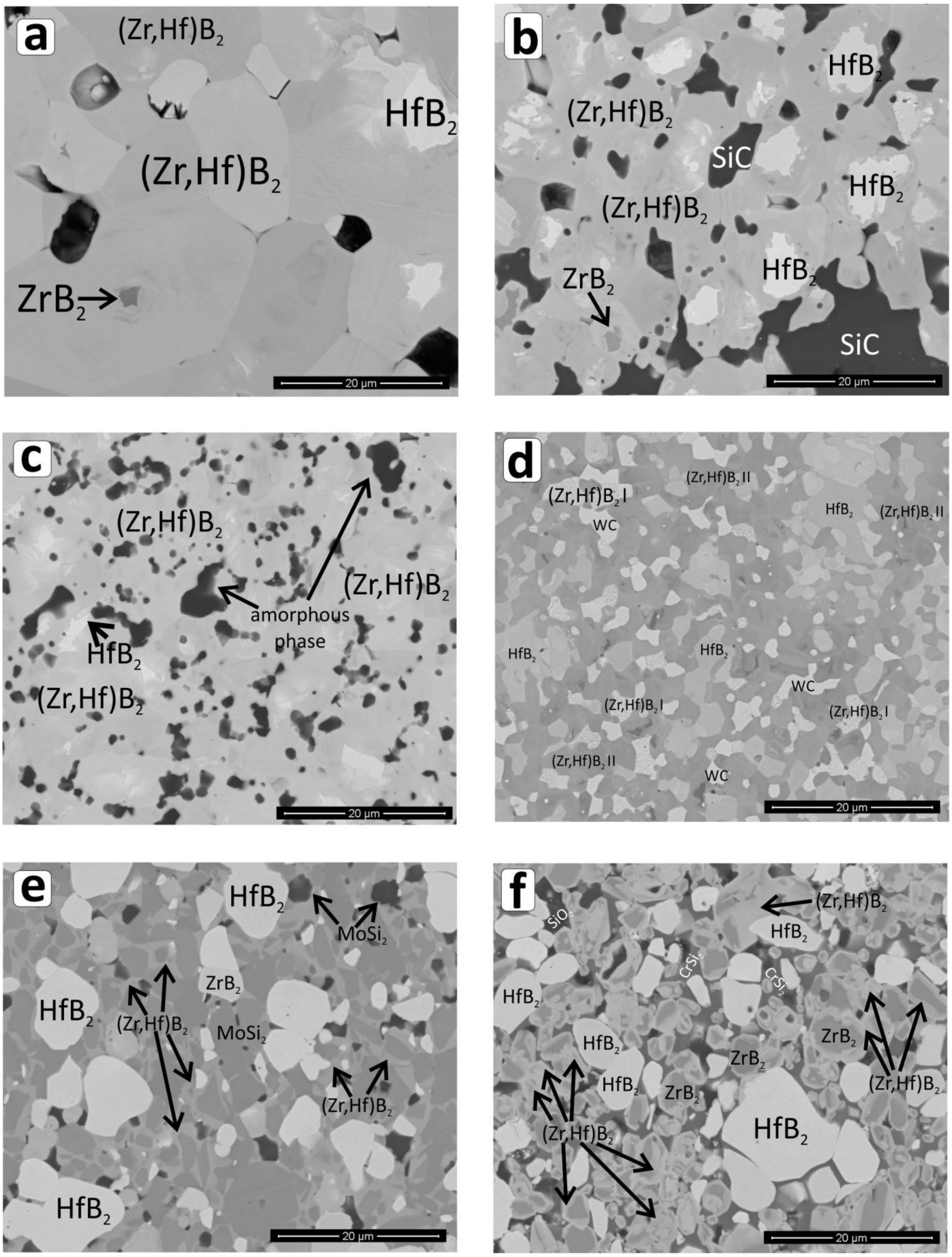


**Figure S4.** SEM microstructures of $ZrB_2$-$HfB_2$ composites sintered by HP: a) without additives, b) with SiC, c) with $B_4C$, d) with WC, e) with $MoSi_2$ f) with $CrSi_2$ additives.

The cross-sections of the oxidated samples at 1400°C are showed in Figure S5.

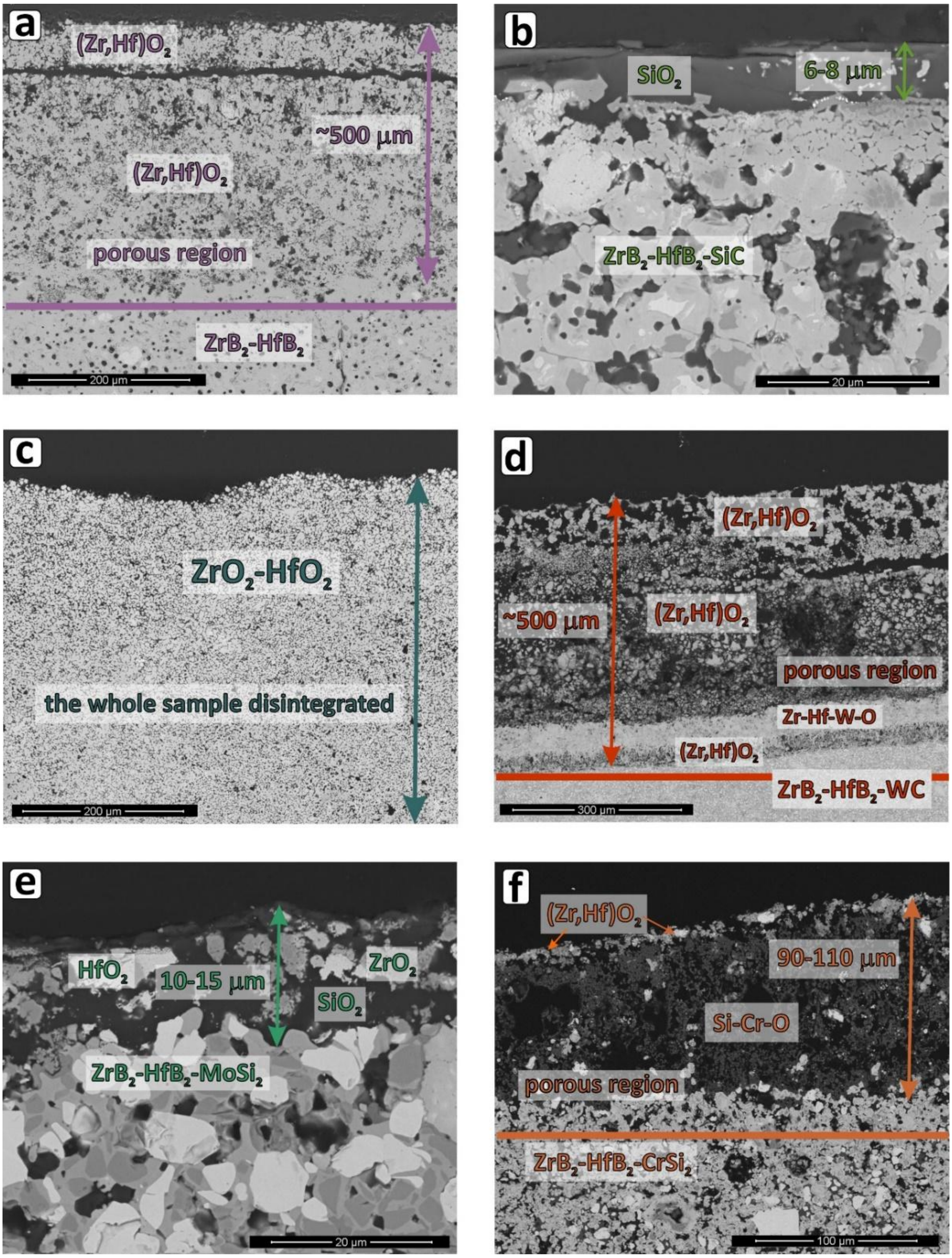


**Figure S5.** Oxidation cross sections of $ZrB_2$-$HfB_2$-MX composites recorded for oxidation temperature 1400 °C: a) HP_0, b) HP_SC; c) HP_BC; d) HP_WC; e) HP_MS i f) HP_CS.

The following **Figures S6 – S11** show elemental distribution maps along the oxidation profiles of all the composites tested.

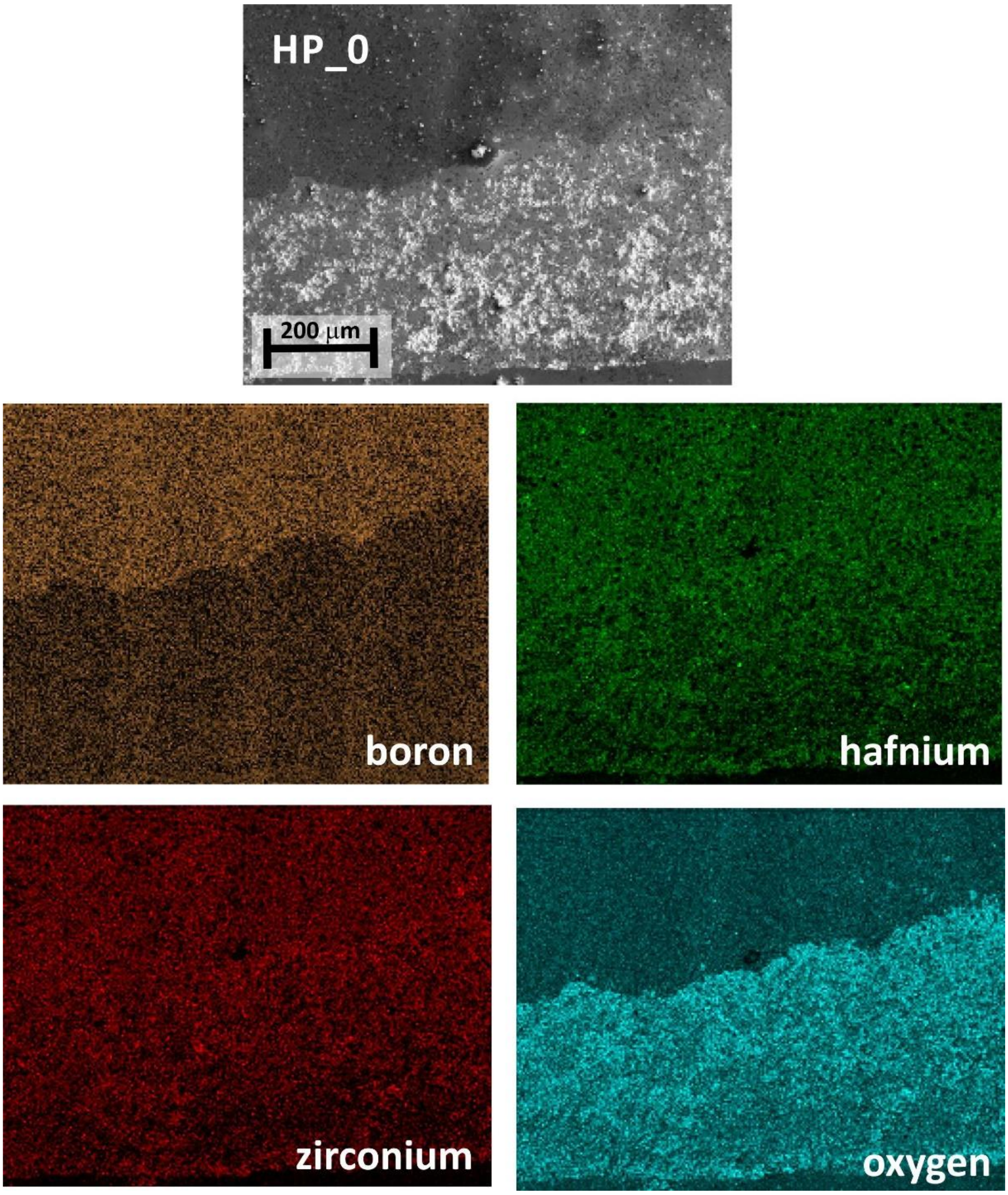


**Figure S6.** Elemental distribution maps along the oxidation profile in HP_0 composite.

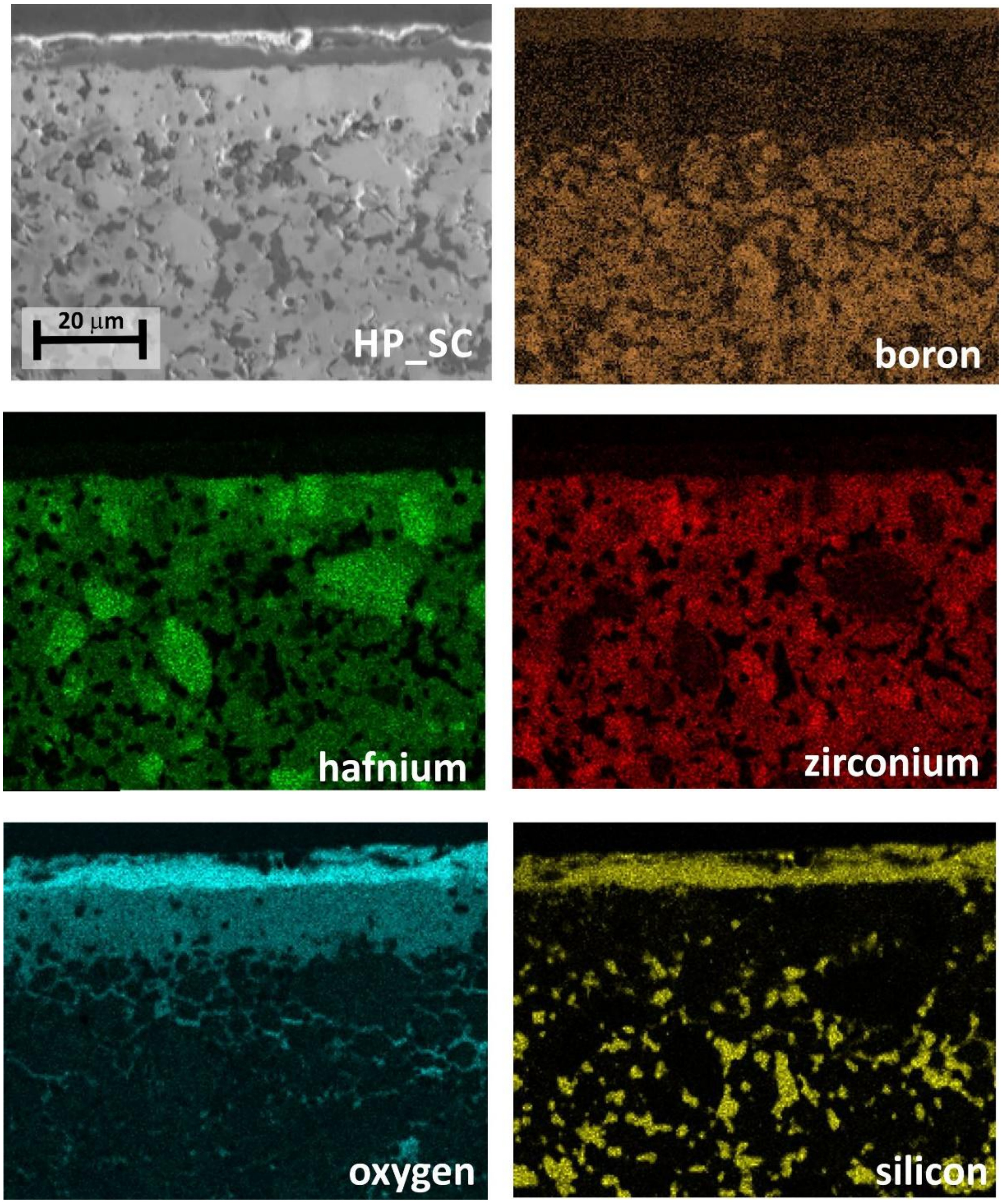

**Figure S7.** Elemental distribution maps along the oxidation profile in the HP_SC composite.

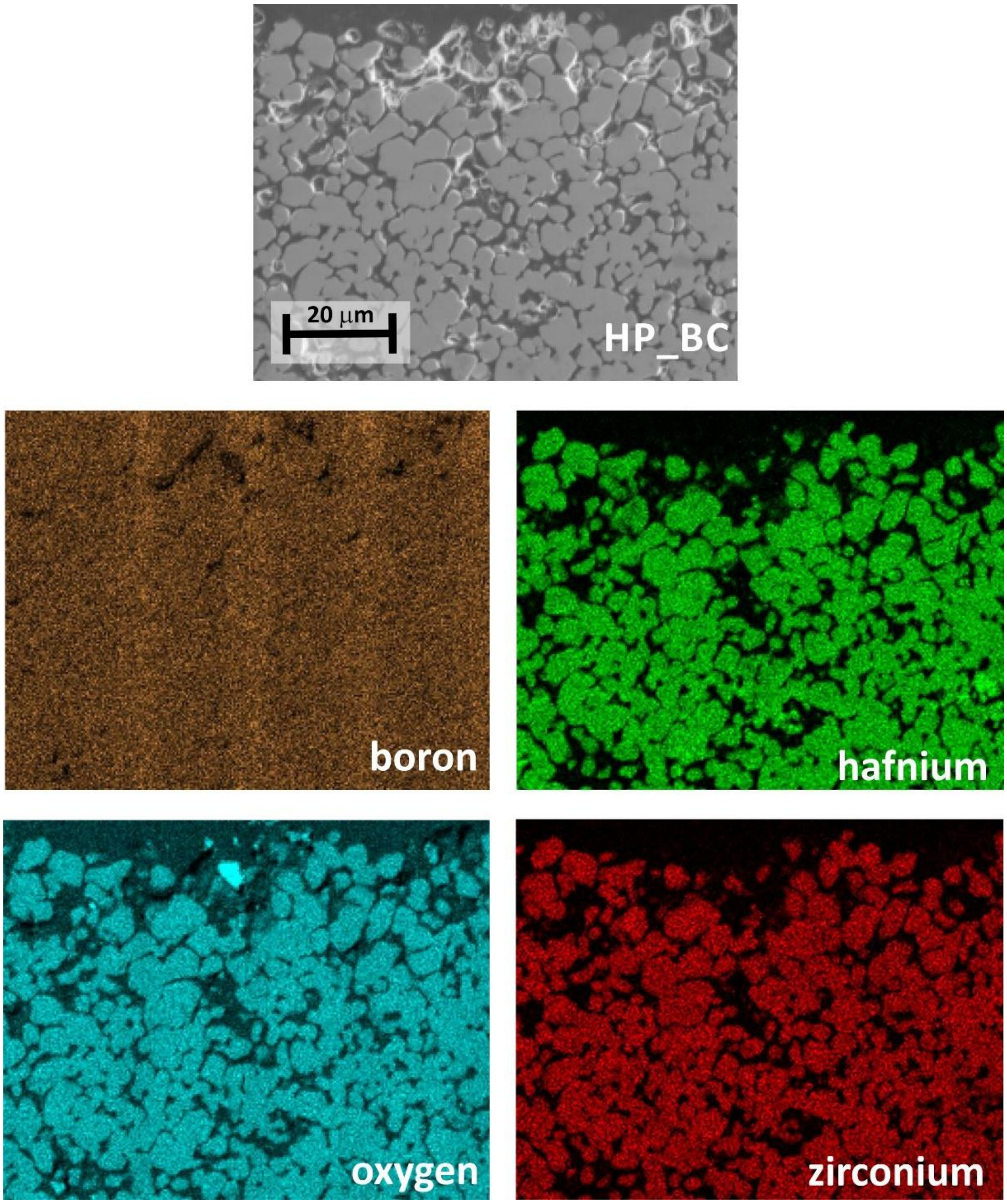

**Figure S8.** Elemental distribution maps along the oxidation profile in the HP_BC composite.

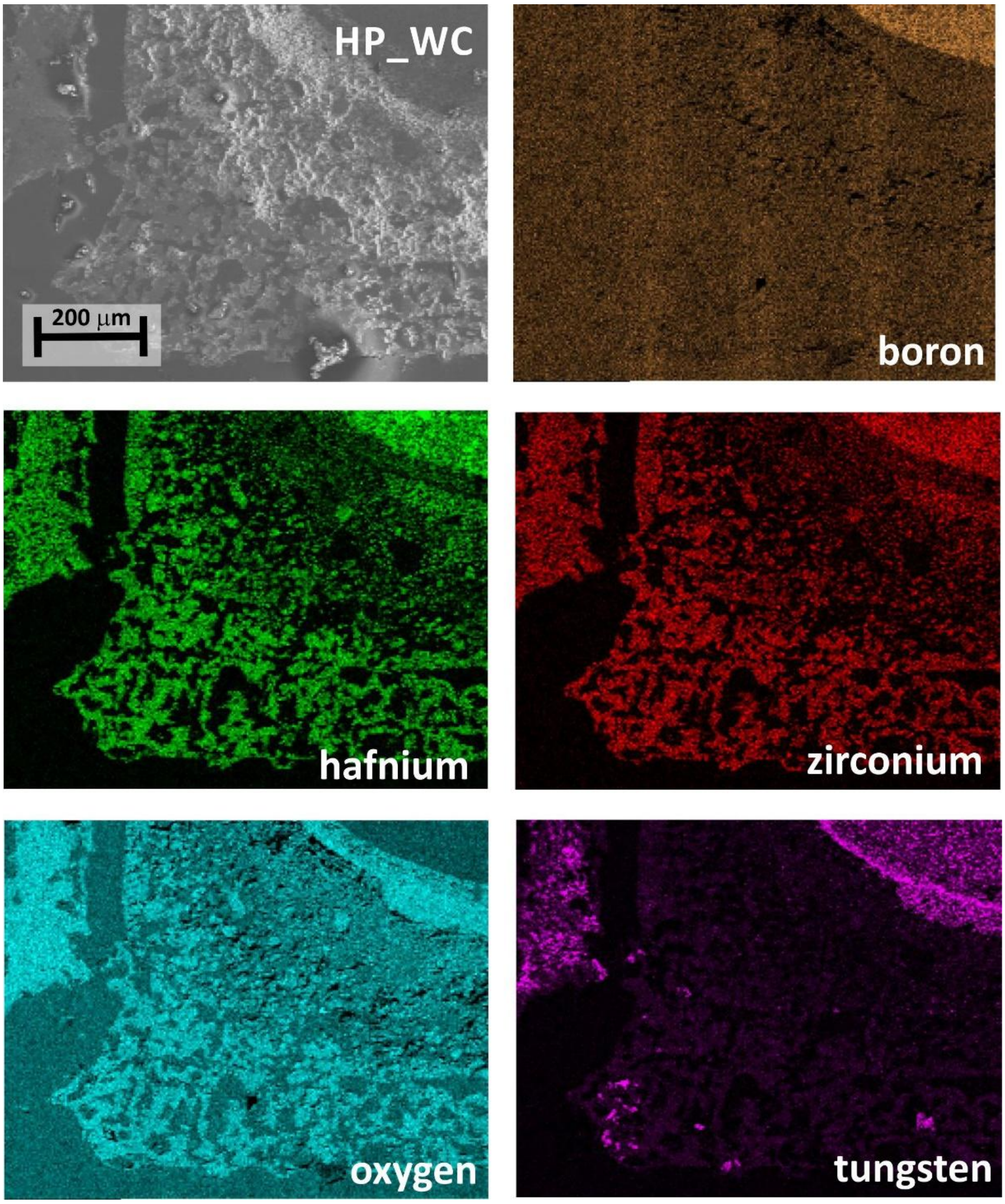

**Figure S9.** Elemental distribution maps along the oxidation profile in HP_WC composite.

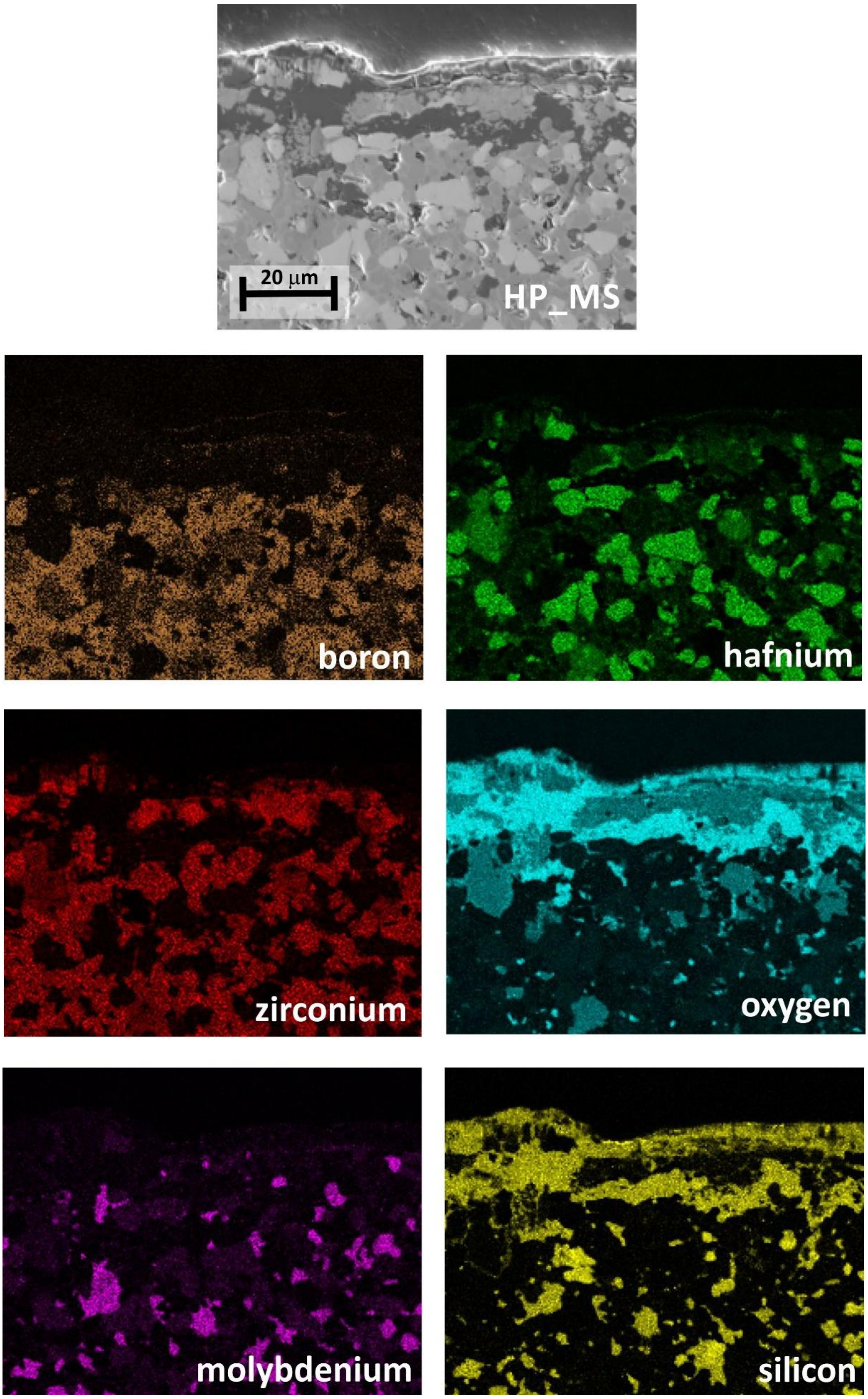


**Figure S10.** Elemental distribution maps along the oxidation profile in the HP_MS composite.

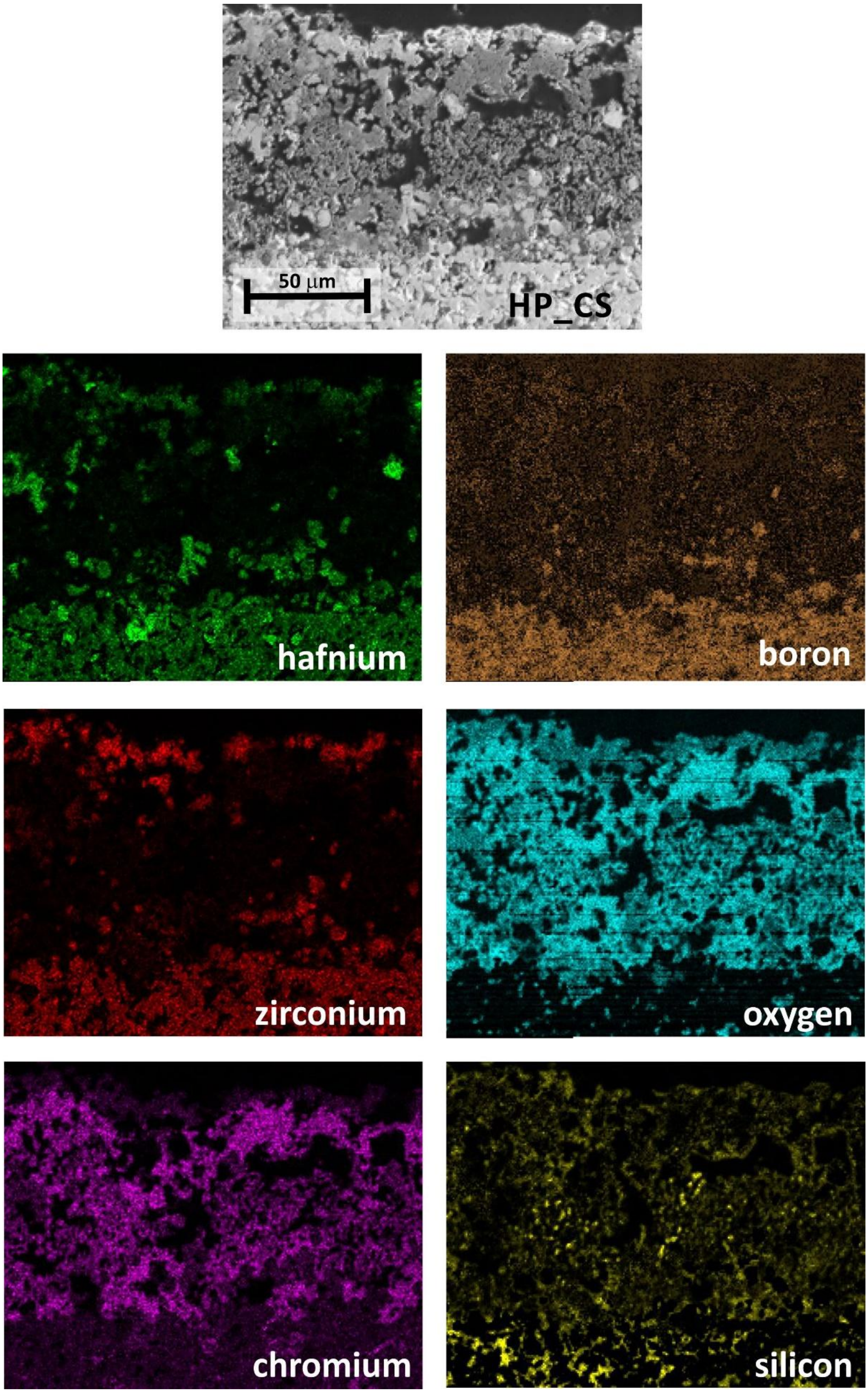


**Figure S11.** Elemental distribution maps along the oxidation profile in the HP_CS composite.
